# Insights into a Multidrug Resistant *Escherichia coli* Pathogen of the Globally Disseminated ST131 Lineage: Genome Analysis and Virulence Mechanisms

**DOI:** 10.1371/journal.pone.0026578

**Published:** 2011-10-28

**Authors:** Makrina Totsika, Scott A. Beatson, Sohinee Sarkar, Minh-Duy Phan, Nicola K. Petty, Nathan Bachmann, Marek Szubert, Hanna E. Sidjabat, David L. Paterson, Mathew Upton, Mark A. Schembri

**Affiliations:** 1 School of Chemistry and Molecular Biosciences, Australian Infectious Diseases Research Centre, University of Queensland, Brisbane, Australia; 2 University of Queensland Centre for Clinical Research, Royal Brisbane and Women's Hospital, Brisbane, Australia; 3 School of Translational Medicine, University of Manchester, Manchester, United Kingdom; Tulane University, United States of America

## Abstract

*Escherichia coli* strains causing urinary tract infection (UTI) are increasingly recognized as belonging to specific clones. *E. coli* clone O25b:H4-ST131 has recently emerged globally as a leading multi-drug resistant pathogen causing urinary tract and bloodstream infections in hospitals and the community. While most molecular studies to date examine the mechanisms conferring multi-drug resistance in *E. coli* ST131, relatively little is known about their virulence potential. Here we examined *E. coli* ST131 clinical isolates from two geographically diverse collections, one representing the major pathogenic lineages causing UTI across the United Kingdom and a second representing UTI isolates from patients presenting at two large hospitals in Australia. We determined a draft genome sequence for one representative isolate, *E. coli* EC958, which produced CTX-M-15 extended-spectrum β-lactamase, CMY-23 type AmpC cephalosporinase and was resistant to ciprofloxacin. Comparative genome analysis indicated that EC958 encodes virulence genes commonly associated with uropathogenic *E. coli* (UPEC). The genome sequence of EC958 revealed a transposon insertion in the *fimB* gene encoding the activator of type 1 fimbriae, an important UPEC bladder colonization factor. We identified the same *fimB* transposon insertion in 59% of the ST131 UK isolates, as well as 71% of ST131 isolates from Australia, suggesting this mutation is common among *E. coli* ST131 strains. Insertional inactivation of *fimB* resulted in a phenotype resembling a slower off-to-on switching for type 1 fimbriae. Type 1 fimbriae expression could still be induced in *fimB*-null isolates; this correlated strongly with adherence to and invasion of human bladder cells and bladder colonisation in a mouse UTI model. We conclude that *E. coli* ST131 is a geographically widespread, antibiotic resistant clone that has the capacity to produce numerous virulence factors associated with UTI.

## Introduction

Clonal dissemination of uropathogenic *Escherichia coli* (UPEC) occurs much more often than is commonly realized [1]. For example, a decade ago, trimethoprim/sulfamethoxazole (TMP/SMX) resistant *E. coli* “clonal group A” was found to be widespread across the United States [2]. Within the last five years, *E. coli* clone O25:H4-ST131 (*E. coli* ST131) has emerged as an important multi-drug resistant extraintestinal pathogen worldwide. Several epidemiological studies have shown *E. coli* ST131 to be a major cause of urinary tract and bloodstream infections within the community as well as in hospitals and long-term care facilities in Europe, Asia, Africa, North America and Australia [3], [4], [5], [6], [7], [8]. In addition, *E. coli* ST131 are also major contributors to what is known as ‘the CTX-M pandemic’; a recent worldwide increase in *E. coli* uropathogens that produce CTX-M type type (‘active on CefoTaXime, first isolated in Munich’) extended spectrum β-lactamases (ESBLs) [9]. *E. coli* ST131 are commonly identified among *E. coli* producing CTX-M-15; currently the most widespread CTX-M ESBL enzyme worldwide [7], [10]. ESBLs mediate resistance to oxyimino-cephalosporins and monobactams but not the cephamycins. Additionally, *E. coli* ST131 is frequently resistant to fluoroquinolones [6], [11]. Therefore, this clone is typically associated with limited treatment options.

Clinical evidence also suggests that pathogens within the ST131 group are highly virulent. Two clinical studies have reported transmission of *E. coli* ST131 strains causing pyelonephritis and septic shock between family members [12], [13]. In addition, phylogenetic analyses of large isolate collections from different geographical locations assign the majority of ST131 strains to phylogenetic group B2, which predominantly comprises virulent extraintestinal *E. coli* strains. Despite this, PCR studies that screened ST131 strains for the presence of several established virulence genes reported the absence of traits commonly associated with B2 phylogeny, particularly adhesins (e.g. P, S and F1C fimbriae) and toxins (e.g. alpha-hemolysin and cytotoxic necrotizing factor 1). In fact only a few virulence genes appear to be uniformly present in strains of the ST131 clone, such as the *fimH* adhesin of type 1 fimbriae, the secreted autotransporter toxin (*sat*), the aerobactin receptor (*iutA*), the uropathogenic-specific protein (*usp*), and the pathogenicity island marker (*malX*) [6], [7], [11], [14]. Thus, our understanding of the virulence capacity of *E. coli* ST131 remains limited, particularly at the level of functional characterisation of virulence determinants. Here we describe the genome sequence of *E. coli* EC958, a multi-drug resistant, phylogenetic group B2, ST131 serotype O25b:H4 strain isolated from the Northwest region of England and use this information to identify and characterise a novel mutation that affects the expression of type 1 fimbriae, a major UPEC virulence factor.

## Results and Discussion

### Genome sequence of *E. coli* strain EC958, a clinical isolate representing ST131 *E. coli* pathogens from the UK

**Figure 1:**
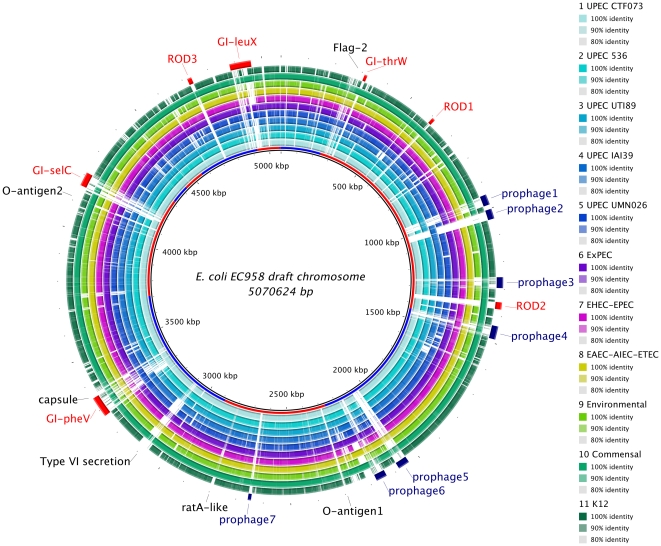
Genomic map of ST131 *E. coli* EC958. The inner circle represents the reference sequence, *E. coli* EC958, with scaffolds of the draft assembly displayed as alternating blue or red color in the inner-most ring. Outer rings show shared identity (according to BLASTn) with individual UPEC genomes and various other *E. coli* genomes. BLASTn matches between 80% and 100% nucleotide identity are colored from lightest to darkest shade, respectively, according to the graduated scale on the right of the circular BLAST image. Matches with less than 80% identity, or *E. coli* EC958 regions with no BLAST matches, appear as blank spaces in each ring. Rings indicate BLAST identity, from inside to out, between EC958 and: (1–5) individual UPEC genomes CFT073, 536, UTI89, IAI39, UMN026 (light to dark blue); (6) pooled ExPEC genomes: APEC O1, IHE3034, S88 (purple); (7) pooled EHEC/EPEC genomes: EC4115, Sakai, EDL933, E2348 (pink); (8) pooled EAEC/AIEC/ETEC genomes: 55989, O42, LF82, E24377A (yellow); (9) environmental *E. coli* genome: SMS_3_5 (lime green); (10) pooled commensal *E. coli* genomes: IAI1, SE11, SE15, HS, 8739, ED1a (teal); pooled *E. coli* K12 genomes: MG1655, W3110, DH10B (green). Black labels indicate regions that are known to be variable in *E. coli* genomes. Blue labels and arcs indicate RODs that appear to be prophage regions; red labels and arcs indicate known genomic islands (GI-*pheV*, GI-*selC*, GI-*thrW*, and GI-*leuX*) or potential new genomic islands (ROD1–3). The image was prepared using Blast Ring Image Generator (http://sourceforge.net/projects/brig).

Fifty four *E. coli* isolates belonging to the ST131 MLST group were collected from patients presenting to both general practice and hospitals in the Northwest region of England, from an area encompassing Greater Manchester in the South to the Lake District in the North (∼150 km South to North). Isolates were recovered during the period of October 2004–June 2009. ST131 strains represent the major pathogenic lineages (PFGE strains A-E) causing UTI across the UK [15]. Strain EC958 was selected as a representative of the ST131 CTX-M-15 producing lineage circulating in the region as it was recovered from a urine sample of a patient presenting in the community with UTI, was gentamicin susceptible, resistant to ciprofloxacin (both characteristic features of ST131 isolates in our collections), produced CTX-M-15 ESBL and carried the CMY-23 type AmpC cephalosporinase [16]. In addition, EC958 is a member of the PFGE defined UK epidemic strain A [15]. A draft genome sequence of EC958 was determined to approximately 29 times coverage. The chromosome was assembled into 14 linear scaffolds with an estimated length of 5,070,614 bp (Figure 1) with two additional circular scaffolds of 133,977 bp and 4088 bp representing a large low-copy and a small high-copy plasmid, respectively. The small plasmid is 97% identical to the 4082 bp pSE11-6 plasmid (dbj|AP009246) identified in the commensal *E. coli* strain SE11. A comparison with complete *E. coli* genomes indicated that EC958 contains several regions of difference (RODs) (Figure 1). These regions are mostly identifiable as prophage elements, with at least 7 distinct loci identified (Figure 1). There are also several loci present in EC958 that are known to be variable across *E. coli* including the Flag-2 lateral flagellar locus, the O-antigen loci, the *ratA*-like toxin encoding gene, the type VI secretion locus and the capsular locus. Comparisons with other UPEC strains (CFT073 [17], 536 [18], UTI89 [19], UMN026 and IAI39 [20]) indicate the presence of four genomic islands in chromosomal integration hot-spots (GI-*pheV*, GI-*selC*, GI-*leuX* and GI-*thrW*, Figure 1). Like the equivalent islands in other UPEC strains, these islands share a modular structure with different combinations and arrangement of virulence-associated genes separated by IS elements [20]. EC958 also carries a 31,448 bp tRNA-*asnT* associated island that shares 99% identity to the High Pathogenicity Island from *Yersinia pestis* [21]. Finally, there are 3 RODs with a length greater than 10 kb with no similarity to known UPEC genomic islands (ROD1–3, Figure 1).

### Antibiotic resistance genes encoded in the pEC958 plasmid

The EC958 genome assembly includes a large plasmid (∼134 kb) with high identity to two previously described ST131 plasmids, pEK499 (from a UK strain A isolate) and pC15-1a, [22], [23]. Plasmid pEC958 is assembled in a single circular scaffold and shares 99–100% identity across 92% of pEK499, including all of the resistance genes found on pEK499 (Table 1). Like other ST131 antibiotic resistance plasmids, antibiotic resistance genes are clustered in cassettes between IS elements (particularly IS*26*), contributing to reassortment and inversions within this region (Table 1). The conjugal transfer region of pEC958 appears to be a composite of pEK499 and pC15-1a. Plasmid pEC958 shares high identity to the pEK499 *tra* region (including the same *traJ* allele) and almost complete synteny in the ∼36 kb between the *rep* and *traC* genes. In contrast to pEK499, pEC958 contains a 22 kb region encoding *traNFQHGSTDIX* conjugal transfer genes that are missing from pEK499 but present in pC15-1a (encompassing the region between *ygeA* and *finO* in pEC958). Notably, pEC958 lacks a small region of *tra* genes (*trbI*, *traW* and *traU*) suggesting that its conjugation apparatus may not be functional, as suggested for pEK499 [22].

**Table 1:** *E. coli* ST131 antibiotic resistance genes.

Gene	Description	pEC958	pEK499^1^	pC15-1a
*bla*_TEM-1_	β-Lactamase TEM-1 precursor	+ (13)^2^	+ (1)	+ (8)
*tet*(A)*tet*(R)	Tetracycline resistance protein, class ATetracycline resistance protein	+ (12)+ (11)	+ (2)+ (3)	+ (1)+ (2)
*aac6′-Ib-cr* *bla*_OXA-1_	Aminoglycoside N(6′)-acetyltransferaseβ-Lactamase OXA-1 precursor	+ (10)+ (9)	+ (4)+ (5)	+ (4)+ (3)
*ΔcatB4* 3	Chloramphenicol acetyltransferase	+ (8)	+ (6)	+ (5)
*aac3′-II*	Aminoglycoside N(3′)-acetyltransferase II	−	−	+ (6)
*bla*_CTX-M-15_	Extended spectrum β-lactamase CTX-M-15	+ (7)	+ (7)	+ (7)
*dhfrVII* *aadA5* *sulI*	Dihydrofolate reductase type VIIAminoglycoside-3-adenyltransferaseDihydropteroate synthetase type I	+ (1)+ (2)+ (3)	+ (8)+ (9)+ (10)	−−−
*mph*(R)*mrx* *mph*(A)	Erythromycin resistance repressor proteinErythromycin resistance regulator proteinMacrolide 2-phosphotransferase	+ (4)+ (5)+ (6)	+ (11)+ (12)+ (13)	−−−
*ampC*(CMY)	AmpC β-Lactamase, CMY-type	−4	−	−

1 +/− indicates presence/absence of indicated gene; Numbers in parentheses refer to order of genes in plasmid; horizontal lines indicate boundaries for antibiotic resistance gene cassettes that are commonly separated by Insertion Sequence (IS) elements.

2 Like in pEK499, the *bla*_TEM-1_ gene in pEC958 is found as part of a complex transposon, separated from the other antibiotic resistance genes by the replication region. In pEC958, however, there is an additional ∼24 kb region encoding conjugation genes between *bla*_TEM-1_ and the replication region.

3 All three plasmids carry a *catB4* gene truncated by an IS26 element at nucleotide position 442 (relative to pEK499 *catB4*). In addition, pC15-1a *catB* has been further disrupted by the inversion of the *aacA4/oxa-1* cassette.

4 An *ampC* gene with 100% amino acid identity to CMY-23 (gb|ABD94117) was identified within a putative genomic island within the EC958 chromosome.

### Virulence factors of EC958

The genome of EC958 contains genes encoding for a variety of potential virulence factors including numerous adhesins, autotransporters, and siderophore receptors (Table 2). These genes correlate well with those reported in recent PCR based studies from the USA [11] and Spain [14], though the current work demonstrates a number of additional virulence genes are present in ST131. In other UPEC strains, several of the virulence factors present in EC958 are found in integrative genomic islands in known chromosomal integration hot-spots. Most important of these appears to be GI-*pheV*, which in EC958 is similar in content and arrangement to the 3′ modules of CFT073 pathogenicity island II including genes for the secreted autotransporter toxin *sat*, an *agn43* homolog and the siderophore receptors *iutA* and *iha* (Table 2). In contrast to the canonical CFT073 *pheV* island, EC958 GI-*pheV* lacks the region between the *int* and *papI* genes and exhibits a translocation and inversion of the *sat*/*iutA* modules [17]. The 51.3 kb GI-*selC* in EC958 shares the same 5′ region with CFT073 GI-*selC*, but is otherwise distinct from other UPEC islands. It carries a complete Dr/Afa chaperone-usher fimbrial operon, which may be important for colonization of the urinary tract. In EC958, the 79.1 kb GI-*leuX* carries the ∼10 kb *fec* siderophore receptor locus found in both UMN026 and IAI39 tRNA-*leuX* islands, but the remaining sequence has not been previously observed in sequenced UPEC strains. At the tRNA-*thrW* chromosomal integration hot-spot, EC958 contains a 10.8 kb genomic island encoding a type I restriction/modification system near-identical to the equivalent region in the B2 commensal *E. coli* O150:H5 strain SE15 [24] but distinct to that in other published UPEC genomes.

**Table 2:** Major virulence factors of EC958 and other sequenced UPEC/ExPEC strains.

	EC958	CFT073	UTI89	536	UMN026	IAI39	IHE3034	Source^1^
**Adhesins**								
*fimA-H* 2	+	+	+	+	+	+	+	CFT073:c5393–5400
*papA-G*	−	+	+	+	+	+	−	CFT073:c3583–3592
*focA-H*	−	+	+	+	−	−	+	CFT073:c1239–1245
*afa*	+	−	−	−	−	−	−	gb|FM955461
*curli*	+	+	+	+	+	+	+	CFT073:c1299–1307
**Autotransporters**								
*agn43* 3	+^4^	+	+^5^	+	+	−	+^5^	CFT073:c1273
*upaG*	+	+	+	+	+^4^	−	+^5^	CFT073:c4424
*upaH*	+	+^4^	+	+	−	−	+	gb|ACX47353
*sat*	+^4^	+	−	−	+	+^4^	−	CFT073:c3619
*picU*	+^5^	+	−	−	−	−	−	CFT073:c0350
**Toxins**								
*hlyA*	−	+	+	+	−	−	−	CFT073:c3570
*cnf1*	−	−	+	−	−	−	−	UTI89:UTI89_C4921
**Iron receptors**								
*fepA*	+	+	+	+	+	+	+	CFT073:c0669
*iroN*	−	+	+	+	−	−	+	CFT073:c1250
*iutA* 2	+^4^	+	−	−	+	+	−	CFT073:c3623
*ireA*	−	+	−	−	−	−	−	CFT073:c5174
*iha* 2	+^4^	+	−	−	+	−	−	CFT073:c3610
*chuA*	+	+	+	+	+	+	+	CFT073:c4308
*hma*	+	+	+	+	−	+	+	CFT073:c2482
*fyuA* 2	+	+	+	+	+	+	+	CFT073:c2436
**Others**								
*kpsM* 2	+	+	+	+^5^	+	+	+	CFT073:c3698
*usp* 2	+	+^4^	+	+	−	−	+	UTI89:UTI89_C0121
*ompT* 2 ^,^ 6	+	+	+	+	+	+	+	CFT073:c0652
*malX* 2	+	+	+	+	+	+	+	CFT073:c2013

1 Strain name and unique locus tag for reference sequence used in BLASTn search of EC958 and complete UPEC genomes (accession number provided for queries not found in complete genome sequences); + or − indicates presence or absence, respectively; unless otherwise indicated, genes share >90% nucleotide identity with reference sequence and presence was confirmed using ACT comparison.

2 Indicated gene found in 91–100% of O25b-ST131 *E. coli* isolates from two previous studies [6], [7].

3 CFT073, UMN026 and 536 have two *agn43* alleles whereas UTI89, IHE3034 and EC958 each have only one; Although the EC958 *agn43* allele is similar to both CFT073 alleles, the UTI89 and IHE3034 alleles are distinct with limited amino acid similarity throughout their central domains (data not shown).

4 Truncated gene or putative pseudogene; in EC958 these are all caused by assembly breaks and may be *bona fide* full-length genes.

5 Gene shares <90% identity with reference sequence.

6 There are two paralogous copies of *ompT* found in IHE3034; CFT073, UTI89, 536 and EC958 carry only a single copy corresponding to IHE3034 ECOK1_0575, whereas UMN026 and IAI39 contain only a single copy corresponding to IHE3034 ECOK1_0571.

In addition, EC958 contains three large regions of difference (RODs 1–3, Figure 1). Interestingly, all share very high identity with syntenic regions in *E. coli* SE15. Both ROD1 and ROD2 contain integrase genes; while ROD2 also contains genes with a predicted sugar transport/metabolism function, the general function of ROD1 genes is unclear. ROD3 carries two tandemly arranged autotransporter homologs (one with 87% nucleotide identity to the 1.2 kb 3′ region of *picU*), a chaperone-usher gene pair and putative fimbrial subunit gene. Given their presence in SE15, these regions may contribute to the fitness of EC958 in gastrointestinal colonisation. Notably, although the backbone of EC958 is most similar to *E. coli* SE15 (including ROD1–3 and GI-*thrW*), the latter strain lacks GI-*pheV*, GI-*selC*, much of GI-*leuX* and six of the prophage identified in EC958.

### Sequence analysis of the type 1 fimbriae encoding genes in *E. coli* EC958

**Figure 2:**
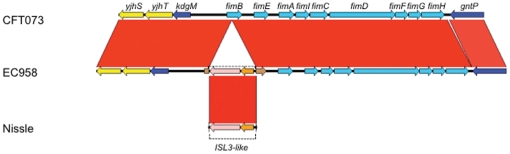
BLASTn comparison of the type 1 fimbriae operon (blue) from *E. coli* strains CFT073, EC958 and a genomic fragment from *Escherichia coli* strain Nissle, a natural *fimB* null strain [**28**], showing an insertion within *fimB*. The red shading indicates high nucleotide identity between the sequences (99–100%). In EC958 the *fimB* gene (brown) has been disrupted by the insertion of an *IS*L3-like transposase gene (light pink) and a gene of unknown function (orange). The inserted sequence, and the point of insertion is nearly identical to that observed in *E. coli* Nissle *fimB* (GenBank:AF188737).

Type 1 fimbriae, encoded by the *fim* genes, are a major virulence factor of UPEC; they mediate binding to α-D-mannosylated proteins such as uroplakins, which are abundant in the uroepithelial lining of the bladder [25], invasion of superficial bladder epithelial cells [26] and the formation of intracellular bacterial communities (IBCs) [27]. Investigation of the *fim* operon sequence in EC958 revealed a 1,895 bp insertion in the *fimB* gene, which encodes the FimB recombinase that switches on the expression of type 1 fimbriae. The insertion in the coding sequence of *fimB* is a single unit containing two coding sequences (CDS) and 23 bp terminal imperfect inverted repeats, flanked by 5 bp direct repeats (Figure 2). One CDS encodes a transposase with sequence similarity to transposases of the ISL*3* family of insertion sequence elements. The second CDS encodes a conserved hypothetical protein with no sequence similarity to transposases or any conserved domains usually associated with transposases.

None of the *E. coli* genomes analysed in Figure 1 contain an insertion in the *fimB* gene. Interestingly, we found that the insertion in EC958 *fimB* is in exactly the same location and almost identical in sequence (with the exception of a 3 bp deletion in one direct repeat and a single SNP) to the *fimB* insertion described in the widely-used probiotic *E. coli* strain Nissle 1917 (Figure 2) [28]. To further investigate the prevalence of the EC958 *fimB* insertion we examined our collection of 54 ST131 clinical isolates by PCR and identified thirty two (59%) that contained an insertion of similar size in *fimB.* Sequencing of the 5′ and 3′ ends of these PCR products revealed that the insertion element among the ST131 strains examined was identical to that identified in the genome of EC958.

### Type 1 fimbriae expression is not a conserved trait among ST131 *E. coli*

We anticipated that inactivation of the FimB recombinase in EC958 would affect the strain's ability to switch on expression of type 1 fimbriae. To test this tenet we used the yeast cell agglutination assay, a standard method for monitoring the production of functional type 1 fimbriae. Overnight shaking cultures of EC958 were negative for type 1 fimbriae expression. As static aerated growth in liquid media strongly selects for expression of type 1 fimbriae, we performed yeast cell agglutination assays with EC958 following three rounds of static culture in LB broth. Static growth resulted in functional expression of type 1 fimbriae in EC958. A similar switching phenotype has been demonstrated for type 1 fimbriae in *E. coli* strain Nissle 1917 [28]. As both strains contain a near identical insertion in the *fimB* gene, it is tempting to speculate that this distinct pattern of slow OFF-to-ON switching of type 1 fimbriae results from the action of another recombinase that can act on the *fim* switch and turn on type 1 fimbriae expression. In the case of the *E. coli* K-12 reference strain MG1655, FimE - the second tyrosine recombinase encoded in the *fim* operon - can switch the *fim* promoter from the ‘off’ to the ‘on’ orientation in the absence of FimB under aerobic static culture conditions [29]. In the *fimB*-null Nissle 1917 strain, however, FimE is not responsible for turning on expression of type 1 fimbriae under static growth conditions, as a double *fimB,fimE* mutant is still able to switch on expression of type 1 fimbriae under these conditions, suggesting that an alternative recombinase mediates off-to-on switching [28]. Two such recombinases, *ipuA* and *ipbA* (also known as *hbiF* and *fimX*) have previously been shown to catalyze off-to-on switching of type 1 fimbriae expression in a *fimBE*-independent manner [30], [31], [32]. The tyrosine recombinase-encoding *ipbA* gene is prevalent among uropathogenic and commensal *E. coli* strains and a copy of the *ipbA* gene is present in the *E. coli* EC958 genome. *E. coli* EC958 also possesses an intact *fimE* gene encoded downstream of *fimB*. Either of these two *fimB* homologs could potentially mediate the observed switching in the absence of *fimB.*

To investigate type 1 fimbriae expression in the ST131 lineage we performed yeast cell agglutination assays for the 54 *E. coli* ST131 isolates in our collection following shaking and static culture in LB broth. Thirty four of the strains (63%) were negative for type 1 expression following shaking culture. Interestingly the majority of these strains (26/34, 76.5%) had a disrupted *fimB* gene due to a sequence insertion, similar to strain EC958. For the ST131 isolates in our collection there was a significant association between encoding a disrupted *fimB* gene and the lack of functional type 1 fimbriae production (*p* = 0.001, Chi-square test). When cultured statically, 27 of the 34 negative strains (79%) became positive for production of type 1 fimbriae, similar to strain EC958, while 7 strains remained negative. Taken together, our screen for type 1 fimbriae expression among ST131 isolates identified three phenotypes: *i)* ‘fim-off’, representing strains unable to switch on expression (7/54, 13%), *ii)* ‘fim-enriched’, representing strains that switch on expression following static growth (27/54, 50%), similar to strain EC958 and *iii)* ‘fim-on’, representing strains that produce type 1 fimbriae under all conditions tested (20/54, 37%). Two strains were selected from each group (Table 3) and compared to the genome-sequenced EC958 (‘fim-enriched’) strain for urovirulence *in vitro* and *in vivo*.

**Table 3:** UK ST131 strains selected for *in vitro* and *in vivo* virulence studies.

Strain ID	Year of isolation	*fimB* insertion	aYA(shaking)	bYA(static)	Type 1 fimbriae phenotype
S15S19	20092009	−−	++	++	fim-onfim-on
S1S27	20072009	+−	−−	−−	fim-offfim-off
EC958S47S52	200520052005	+++	−−−	+++	fim-enrichedfim-enrichedfim-enriched

a Yeast agglutination (YA) following shaking growth.

b Yeast agglutination (YA) following 3 rounds of static growth.

### Virulence properties of *E. coli* EC958 and ST131 *E. coli*

**Figure 3:**
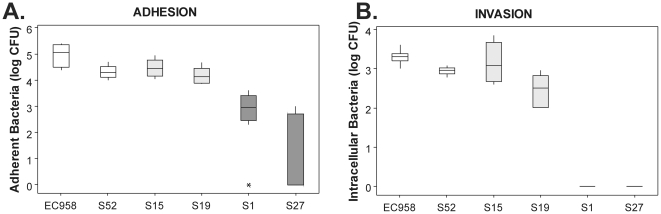
*E. coli* EC958 and ST131 strain adhesion (A) and invasion (B) of human bladder epithelial cells. Triplicate T24 cell monolayers were infected with ST131 strains EC958, and S52 (‘fim-enriched’, white), S15 and S19 (‘fim-on’, light grey), S1 and S27 (‘fim-off’, dark grey) at a multiplicity of infection (MOI) of 10 and incubated at 37°C, 5% CO_2_ for 1 hour. Adherent and intracellular bacteria were enumerated by plating on LB agar. Boxplots summarize CFU/ml data of three experimental repeats.

The selected *E. coli* ST131 strains were tested for virulence phenotypes that are commonly associated with uropathogenesis. Bacterial adhesion of six *E. coli* ST131 strains to host cells was assessed using T24 human bladder epithelial cell monolayers. After overnight static culture to enrich for type 1 fimbriae the ‘fim-enriched’ strains (EC958 and S52) and the ‘fim-on’ strains (S15 and S19) were highly adherent to bladder epithelial cells, while the ‘fim-off’ strains (S1 and S27) bound significantly less (P<0.001, Kruskal-Wallis; Figure 3A). The adherent strains were also able to invade T24 bladder epithelial cells, while the intracellular loads of the two ‘fim-off’ strains were significantly lower (P<0.001, Kruskal-Wallis; Figure 3B).

### Type 1 fimbriae expression in *E. coli* EC958 is required for colonisation of the bladder, but not persistence in urine, in a mouse infection model

**Figure 4:**
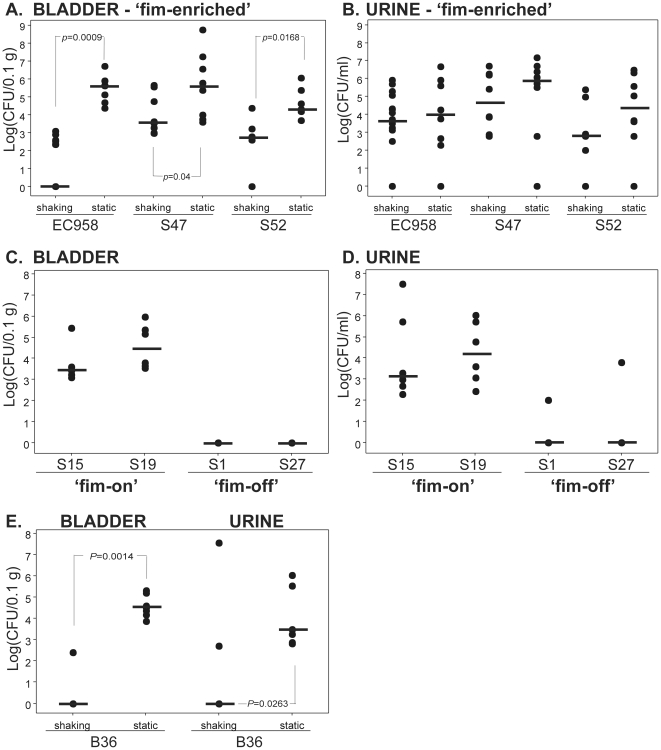
Mouse urinary tract colonisation by *E. coli* EC958 and ST131 strains. A minimum of eight C57BL/6 mice were transurethrally inoculated with ∼5×10^8^ CFU of each ST131 strain. After 18 h urine and bladder homogenate samples were plated on LB agar in triplicate for determination of bacterial loads. Data are presented as log CFU per ml of urine or 0.1 g of tissue. Bladder (A) and urine (B) bacterial loads of ‘fim-enriched’ ST131 strains EC958, S47 and S52 determined following shaking or static growth of the culture inoculum. Bladder (C) and urine (D) bacterial loads of S15, S19 (‘fim-on’) and S1, S27 (‘fim-off’) ST131 strains. (E) Bladder and urine bacterial loads of B36 following shaking and static growth. B36 is an ST131 ‘fim-enriched’ strain from our Australian collection. Equality of group medians was tested using the Mann-Whitney nonparametric test. Shown P values<0.05 are considered significant.

Our finding that *fimB* is disrupted in strain EC958 and many of the ST131 isolates in our collection, together with the effects of the insertion on the production of type 1 fimbriae, prompted us to investigate the ability of ST131 strains to colonise the mouse urinary tract. We used the mouse model of ascending UTI to test the colonisation potential of EC958 where the inoculum was cultured under shaking (low expression of type 1 fimbriae) or static (high expression of type 1 fimbriae) conditions. EC958 failed to colonise the mouse bladder after shaking culture, but upon expression of type 1 fimbriae (static culture) bladder colonisation increased dramatically by a significant 5.6 log median difference in CFU/0.1 g tissue (*p*<0.001, Mann-Whitney) (Figure 4A). In contrast, EC958 bacterial counts in urine were high following both shaking and static culture (Figure 4B). To investigate the importance of this finding further, we extended our *in vivo* study to include two other ‘fim-enriched’ ST131 isolates (S47 and S52). Similar to our results with EC958, bladder colonisation by S47 and S52 was significantly enhanced after static culture (*p*<0.05, Mann-Whitney) (Figure 4A). In addition, both strains were successfully recovered from mouse urine and the numbers were not significantly different between shaking and static culture conditions (Figure 4B). As a comparison, we also examined the colonisation potential of two ‘fim-on’ (S15 and S19) and two ‘fim-off’ (S1 and S27) ST131 isolates *in vivo*. The ‘fim-on’ strains were able to colonise the mouse bladder and urine at levels comparable to the ‘fim-enriched’ strains, while the ‘fim-off’ strains were recovered in significantly lower numbers from mouse bladders and urine (Figure 4C–D). The molecular mechanism that defines the ‘fim-off’ state, as well as the mechanism by which these strains can colonize the human bladder and cause disease, remains unknown. Taken together our results demonstrate that expression of type 1 fimbriae enhances bladder colonisation by ST131 strains with the ‘fim-enriched’ switching phenotype, but strong expression of type 1 fimbriae is not required for EC958 persistence in urine *in vivo*.

### An *E. coli* EC958 *fim* mutant is attenuated for colonisation of the mouse bladder

**Figure 5:**
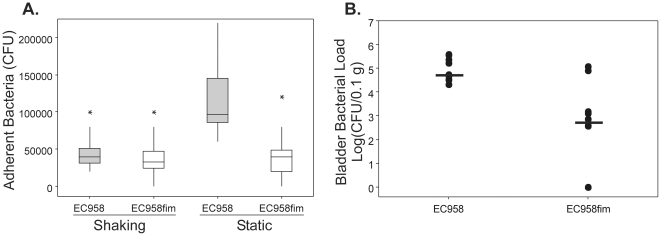
Virulence of *E. coli* EC958 and isogenic *fimD* knockout mutant. (A) Adhesion of EC958 (grey) and EC958*fimD* (white) to T24 human bladder epithelial cells following shaking or static culture. Boxplots summarize CFU/ml data of three experimental repeats. (B) Bacterial loads in mouse bladders recovered 18 hours post- infection with EC958 and EC958*fimD*. Data are presented for each mouse as log_10_ (CFU/0.1 g tissue). Horizontal bars show group medians.

In order to dissect the contribution of type 1 fimbriae to *E. coli* ST131 virulence we constructed an isogenic *fimD* knockout mutant in EC958. To overcome the challenge of genetically manipulating the multidrug resistant EC958 strain, we modified the *λ*-Red mediated homologous recombination method [33] by cloning the gentamicin resistance gene into a vector containing the *λ*-Red recombinase. Insertional inactivation of *fimD* was performed using the chloramphenicol resistance cassette from pKD3, since the native *catB4* gene encoded in pEC958 is truncated by an IS26 element (see Table 2). The isogenic EC958*fimD* mutant lacked surface expression of type 1 fimbriae following either shaking or static culture and was unable to adhere in significant numbers to T24 bladder epithelial cells (*p*<0.001, Figure 5A). The ability of EC958*fimD* to colonise the mouse bladder was compared to wild-type EC958 in a mouse model of UTI. EC958*fimD* was recovered from the mouse bladder in significantly reduced numbers compared to the wild-type EC958 strain (*p*<0.05, Mann-Whitney) (Figure 5B). The same result was also observed in 1∶1 mixed infection experiments, where EC958 significantly outcompeted EC958*fimD* for bladder colonisation (Log_10_ competitive index [EC958*fimD*/EC958] = −3.426; *p* = 0.003, Wilcoxon Signed Rank). Our findings using the EC958*fimD* knockout strain demonstrate a role for type 1 fimbriae in bacterial adhesion and bladder colonisation. Taken together our results demonstrate that the genome-sequenced ST131 strain EC958 has a distinct set of virulence phenotypes and *in vivo* colonisation pattern that are also shared with other ‘fim-enriched’ strains from the UK ST131 isolate collection.

### The insertion in *fimB* in EC958 is also present in *E. coli* isolates of the ST131 lineage from Australia

We extended our study to a collection of twenty one ST131 isolates from UTI patients presenting to two hospitals in Australia from 2007–2010. We detected the same insertion in *fimB* in 71% of these isolates. The sequence was highly identical to that present in EC958 and type 1 fimbriae expression in these isolates displayed the same ‘fim-enriched’ phenotype observed with EC958 following static culture conditions. Most importantly, a similar pattern of *in vivo* bladder colonisation to that of EC958 was observed with B36, one of the ‘fim-enriched’ isolates from Australia that was tested in the mouse UTI model (Figure 4E). These results provide further support for a role of type 1 fimbriae in bladder colonisation by isolates belonging to the ST131 lineage.

### Conclusions

The rapid and widespread dissemination of multiply antibiotic resistant UPEC strains from the ST131 lineage in hospitals and the community represents a serious threat to healthcare resources worldwide. Alarmingly, ST131 strains that have acquired a new type of metallo-β-lactamase, NDM-1, conferring resistance to carbapenems - often our last line of defence against multiresistant *E. coli* infections - have been recently reported [34]. The identification of NDM-1 producing ST131 strains highlights the importance of understanding the virulence mechanisms employed by this highly successful *E. coli* clone. This study provides the first genomic insight into this emerging group of *E. coli* pathogens and reveals the extent of its virulence potential. Even though the strain is particularly well equipped to resist the action of many antibiotics, the genome sequence of *E. coli* ST131 strain EC958 contains an extensive array of UPEC-associated virulence factors, genomic islands and prophage regions that distinguish it from other *E. coli* pathotypes. It has previously been suggested that carriage of the genes required for both pronounced virulence and high level antibiotic resistance results in strains that are metabolically disadvantaged and not fit for persistence/infection. This is clearly not the case for ST131 strains. *E. coli* EC958 carries a CTX-M15 plasmid which is a cointegrate of pEK499 and pC15-1a with resistance to eight antibiotic classes. Sequencing the genome of EC958 facilitated the identification of type 1 fimbriae as a key virulence factor in its pathogenicity. The transposase insertion identified in the *fimB* recombinase in the genome of EC958 was also found in multiple independently isolated ST131 strains from opposite sides of the globe, suggesting that it occurred early in the divergence of the ST131 lineage. Using *in vitro* functional assays we demonstrated that this insertion results in reduced frequency of switching on type 1 fimbriae expression and implicates the activity of a second tyrosine recombinase in this process. Two candidate recombinases have been identified in the genome of EC958 and studies investigating their role in switching on type 1 fimbriae expression are under way. Using a mouse model of ascending UTI we established the role of type 1 fimbriae in EC958 virulence *in vivo*. Sequencing more *E. coli* ST131 clinical isolates from geographically diverse collections will add to our understanding of the genetic basis for uropathogenesis and the global dissemination of strains belonging to the *E. coli* ST131 clonal group.

## Materials and Methods

### Ethical approval

This study was carried out in strict accordance with the recommendations in the Animal Care and Protection Act (Queensland, 2002) and the Australian Code of Practice for the Care and Use of Animals for Scientific Purposes (7th edition, 2004). Approval for mouse infection studies was obtained from the University of Queensland Animal Ethics Committee (SCMB/471/09/NHMRC (NF)).

### Strains

Clinical UTI isolates were obtained from urine samples received by microbiology laboratories at hospitals in Manchester and Preston, Northwest England and Brisbane, Australia. *E. coli* isolates were collected as part of routine methods for the diagnosis of UTI. All samples were de-identified; individual informed consent was not required.

All *E. coli* strains used in this study were routinely cultured at 37°C on solid or in liquid Luria Broth (LB) medium. All ST131 isolates met the selection criteria of being recovered from non-duplicate urine cultures of patients presenting with UTI, where CFU counts were >1×10^5^ ml^−1^ with the presence of elevated levels of white cells.

### Molecular methods

The isolates examined in the current study were subjected to multilocus sequence typing (MLST) as previously described [15], [35]. Some of the isolates in the current collection were included in these previous studies. Chromosomal DNA purification, PCR and DNA sequencing of PCR products was performed as previously described [36]. PCR screening and sequencing of the *fimB* insertion was performed using oligonucleotides fimB-IS_F 5′-TCCTGACCCATAGTGAAATCG-3′ and fimB-IS_R 5′-GCTCTATCCCAGATGCCGTA-3′. For insertional inactivation of the *fimD* gene in EC958, the chloramphenicol resistance cassette was amplified from pKD3 using primers 2141 (5′- cgcaactcgccagtatggggctgaatatggcttctgtcgccggtatgaatctgctggcggatgatgcctgtgggaattagccatggtcc) and 2142 (5′-gtatccagcgccactctgttttcccgatattcggtggcataaggcagcacggcataaccgcgccagtcgggtgtaggctggagctgcttc), containing 50 nucleotide flanking regions complementary to the beginning and end of *fimD*. The knock-out PCR product was introduced by electroporation into EC958 transformed with a gentamicin resistant plasmid carrying the *λ*-Red recombinase. Insertional inactivation of *fimD* was performed as previously described [33] and the constructed mutant was confirmed by sequencing of the insertion sites.

### Genome sequencing and annotation of *E. coli* EC958

Genomic DNA of *E. coli* EC958 was sequenced using a 454 GS-FLX by the Centre for Genomic Research, University of Liverpool. The 454 GS Assembler 2.5.3 software was used to assemble 826269 sequence reads into 240 contigs greater than 200 bp in length with an average depth of 29-fold coverage. Approximately half of the reads were mate-paired (with an average insert size of 3189) enabling 155 contigs to be linked into 16 scaffolds. Chromosomal scaffolds ranged in size from 2,462 bp to 1,197,002 bp with 99% of the genome assembled into 9 large scaffolds greater than 100 kb in length. After manual validation of the assembly using consed [37], scaffolds were ordered according to the genome of UPEC UTI89 [19] and annotated using SUGAR, an in-house draft genome annotation pipeline (Szubert and Beatson, unpublished). The assembly also included 175 unscaffolded contigs (>200 bp) corresponding mostly to repetitive elements such as insertion sequences (IS) and rRNA operons. Comparison and visualization of *E. coli* genomes was carried out using BLAST [38], ACT [39], Easyfig [40] and BRIG [41]. The EC958 draft scaffolds have been deposited in the EMBL WGS database (EMBL Accession CAFL01000001 to CAFL01000240). The features of the noted regions from the EC958 genome (Figure 1) are provided in Table S1. The sequence of each of these regions is provided in Dataset S1.

### Yeast cell agglutination assay

Expression of functional type 1 fimbriae on the surface of ST131 *E. coli* strains was determined by agglutination of yeast cells (*Saccharomyces cerevisiae*). Clinical isolates were plated onto LB agar. Five single colonies of each strain were pooled, inoculated into 3 ml LB and incubated shaking or statically for 24 h. Static cultures were then passaged statically in two further rounds of incubation. Agglutination was performed by mixing a suspension of bacterial cells with an equal volume of yeast cells (diluted to 5% in phosphate buffered saline [PBS]) on a glass slide and the visual observation of bacterial-yeast aggregation. UPEC strains UTI89 and 83972 were used as positive and negative controls in all assays. ST131 strains that failed to produce visible agglutination of yeast cells after 3 minutes were scored negative.

### Epithelial cell adhesion and invasion assay

The ability of ST131 strains to adhere to human bladder epithelial cells was tested as previously described [42]. T24 bladder epithelial cells (ATCC HTB-4) were maintained in McCoy's 5A medium (modified) (Invitrogen) supplemented with 10% heat-inactivated fetal calf serum (Invitrogen). Confluent T24 cell monolayers were infected with ST131 strains at a multiplicity of infection (MOI) of 1∶10 and incubated at 37°C, 5% CO_2_ for 1 hour. Non-adherent bacteria were removed by 5 washes with PBS. T24 cell lysates were serially diluted and plated onto LB agar plates to enumerate adherent bacteria. Enumeration of intracellular bacterial loads was performed in a similar way, following exclusion of extracellular bacteria by gentamicin treatment (200 µg ml^−1^) for 1 hour.

### Mouse model of UTI

The C57BL/6 mouse model of ascending UTI was employed as previously described [36]. Briefly, female C57BL/6 mice (8–10 weeks) were transurethrally inoculated with ∼5×10^8^ CFU using a 1 ml tuberculin syringe attached to a sterile catheter. After 18 hours, urine and homogenated bladder samples were processed for bacterial loads by viable CFU counts performed in triplicate. Data are presented as log CFU per ml of urine or 0.1 g of tissue for each mouse. A minimum of 8 mice was included in each strain group. Equality of group medians was tested using the Mann-Whitney nonparametric test.

## Supporting Information

Table S1 **Features of the specific regions from the EC958 genome noted in** **Figure 1** **.**
(XLSX)

Dataset S1 **Sequence of each of the specific regions from the EC958 genome noted in Table S1.**
(TXT)

## References

[1] Stamm WE (2001). An epidemic of urinary tract infections?. N Engl J Med.

[2] Manges AR, Johnson JR, Foxman B, O'Bryan TT, Fullerton KE (2001). Widespread Distribution of Urinary Tract Infections Caused by a Multidrug-Resistant Escherichia coli Clonal Group.. New England Journal of Medicine.

[3] Platell JL, Cobbold RN, Johnson JR, Trott DJ (2010). Clonal group distribution of fluoroquinolone-resistant Escherichia coli among humans and companion animals in Australia.. J Antimicrob Chemother.

[4] Sidjabat HE, Derrington P, Nimmo GR, Paterson DL (2010). Escherichia coli ST131 producing CTX-M-15 in Australia.. J Antimicrob Chemother.

[5] Cagnacci S, Gualco L, Debbia E, Schito GC, Marchese A (2008). European emergence of ciprofloxacin-resistant Escherichia coli clonal groups O25:H4-ST 131 and O15:K52:H1 causing community-acquired uncomplicated cystitis.. J Clin Microbiol.

[6] Johnson JR, Menard M, Johnston B, Kuskowski MA, Nichol K (2009). Epidemic clonal groups of Escherichia coli as a cause of antimicrobial-resistant urinary tract infections in Canada, 2002 to 2004.. Antimicrob Agents Chemother.

[7] Nicolas-Chanoine MH, Blanco J, Leflon-Guibout V, Demarty R, Alonso MP (2008). Intercontinental emergence of Escherichia coli clone O25:H4-ST131 producing CTX-M-15.. J Antimicrob Chemother.

[8] Pitout JD, Gregson DB, Campbell L, Laupland KB (2009). Molecular characteristics of extended-spectrum-beta-lactamase-producing Escherichia coli isolates causing bacteremia in the Calgary Health Region from 2000 to 2007: emergence of clone ST131 as a cause of community-acquired infections.. Antimicrob Agents Chemother.

[9] Canton R, Coque TM (2006). The CTX-M beta-lactamase pandemic.. Curr Opin Microbiol.

[10] Coque TM, Novais A, Carattoli A, Poirel L, Pitout J (2008). Dissemination of clonally related Escherichia coli strains expressing extended-spectrum beta-lactamase CTX-M-15.. Emerg Infect Dis.

[11] Johnson JR, Johnston B, Clabots C, Kuskowski MA, Castanheira M (2010). Escherichia coli sequence type ST131 as the major cause of serious multidrug-resistant E. coli infections in the United States.. Clin Infect Dis.

[12] Ender PT, Gajanana D, Johnston B, Clabots C, Tamarkin FJ (2009). Transmission of an Extended-Spectrum-Beta-Lactamase-Producing Escherichia coli (Sequence Type ST131) Strain between a Father and Daughter Resulting in Septic Shock and Emphysematous Pyelonephritis.. J Clin Microbiol.

[13] Johnson JR, Anderson JT, Clabots C, Johnston B, Cooperstock M (2009). Within-Household Sharing of a Fluoroquinolone-Resistant Escherichia Coli Sequence Type St131 Strain Causing Pediatric Osteoarticular Infection.. Pediatr Infect Dis J.

[14] Coelho A, Mora A, Mamani R, Lopez C, Gonzalez-Lopez JJ (2011). Spread of Escherichia coli O25b:H4-B2-ST131 producing CTX-M-15 and SHV-12 with high virulence gene content in Barcelona (Spain).. J Antimicrob Chemother.

[15] Lau SH, Kaufmann ME, Livermore DM, Woodford N, Willshaw GA (2008). UK epidemic *Escherichia coli* strains A–E, with CTX-M-15 {beta}-lactamase, all belong to the international O25:H4-ST131 clone.. J Antimicrob Chemother.

[16] Woodford N, Reddy S, Fagan EJ, Hill RL, Hopkins KL (2007). Wide geographic spread of diverse acquired AmpC beta-lactamases among Escherichia coli and Klebsiella spp. in the UK and Ireland.. J Antimicrob Chemother.

[17] Welch RA, Burland V, Plunkett G, Redford P, Roesch P (2002). Extensive mosaic structure revealed by the complete genome sequence of uropathogenic Escherichia coli.. Proc Natl Acad Sci U S A.

[18] Brzuszkiewicz E, Bruggemann H, Liesegang H, Emmerth M, Olschlager T (2006). How to become a uropathogen: comparative genomic analysis of extraintestinal pathogenic Escherichia coli strains.. Proc Natl Acad Sci U S A.

[19] Chen SL, Hung CS, Xu J, Reigstad CS, Magrini V (2006). Identification of genes subject to positive selection in uropathogenic strains of Escherichia coli: a comparative genomics approach.. Proc Natl Acad Sci U S A.

[20] Touchon M, Hoede C, Tenaillon O, Barbe V, Baeriswyl S (2009). Organised genome dynamics in the Escherichia coli species results in highly diverse adaptive paths.. PLoS Genet.

[21] Gehring AM, DeMoll E, Fetherston JD, Mori I, Mayhew GF (1998). Iron acquisition in plague: modular logic in enzymatic biogenesis of yersiniabactin by Yersinia pestis.. Chem Biol.

[22] Woodford N, Carattoli A, Karisik E, Underwood A, Ellington MJ (2009). Complete nucleotide sequences of plasmids pEK204, pEK499, and pEK516, encoding CTX-M enzymes in three major Escherichia coli lineages from the United Kingdom, all belonging to the international O25:H4-ST131 clone.. Antimicrob Agents Chemother.

[23] Boyd DA, Tyler S, Christianson S, McGeer A, Muller MP (2004). Complete nucleotide sequence of a 92-kilobase plasmid harboring the CTX-M-15 extended-spectrum beta-lactamase involved in an outbreak in long-term-care facilities in Toronto, Canada.. Antimicrob Agents Chemother.

[24] Toh H, Oshima K, Toyoda A, Ogura Y, Ooka T (2010). Complete genome sequence of the wild-type commensal Escherichia coli strain SE15, belonging to phylogenetic group B2.. J Bacteriol.

[25] Wu XR, Sun TT, Medina JJ (1996). In vitro binding of type 1-fimbriated Escherichia coli to uroplakins Ia and Ib: relation to urinary tract infections.. Proc Natl Acad Sci U S A.

[26] Martinez JJ, Mulvey MA, Schilling JD, Pinkner JS, Hultgren SJ (2000). Type 1 pilus-mediated bacterial invasion of bladder epithelial cells.. Embo J.

[27] Anderson GG, Palermo JJ, Schilling JD, Roth R, Heuser J (2003). Intracellular bacterial biofilm-like pods in urinary tract infections.. Science.

[28] Stentebjerg-Olesen B, Chakraborty T, Klemm P (1999). Type 1 fimbriation and phase switching in a natural Escherichia coli fimB null strain, Nissle 1917.. J Bacteriol.

[29] Stentebjerg-Olesen B, Chakraborty T, Klemm P (2000). FimE-catalyzed off-to-on inversion of the type 1 fimbrial phase switch and insertion sequence recruitment in an Escherichia coli K-12 fimB strain.. FEMS Microbiol Lett.

[30] Bryan A, Roesch P, Davis L, Moritz R, Pellett S (2006). Regulation of type 1 fimbriae by unlinked FimB- and FimE-like recombinases in uropathogenic Escherichia coli strain CFT073.. Infect Immun.

[31] Hannan TJ, Mysorekar IU, Chen SL, Walker JN, Jones JM (2008). LeuX tRNA-dependent and -independent mechanisms of Escherichia coli pathogenesis in acute cystitis.. Molecular microbiology.

[32] Xie Y, Yao Y, Kolisnychenko V, Teng CH, Kim KS (2006). HbiF regulates type 1 fimbriation independently of FimB and FimE.. Infection and immunity.

[33] Datsenko KA, Wanner BL (2000). One-step inactivation of chromosomal genes in Escherichia coli K-12 using PCR products.. Proc Natl Acad Sci U S A.

[34] Peirano G, Schreckenberger PC, Pitout JD (2011). The characteristics of NDM-1-producing Escherichia coli that belong to the successful and virulent clone ST131.. Antimicrob Agents Chemother.

[35] Lau SH, Reddy S, Cheesbrough J, Bolton FJ, Willshaw G (2008). Major Uropathogenic Escherichia coli Strain Isolated in the Northwest of England Identified by Multilocus Sequence Typing.. J Clin Microbiol.

[36] Allsopp LP, Totsika M, Tree JJ, Ulett GC, Mabbett AN (2010). UpaH is a newly identified autotransporter protein that contributes to biofilm formation and bladder colonization by uropathogenic Escherichia coli CFT073.. Infect Immun.

[37] Gordon D, Abajian C, Green P (1998). Consed: a graphical tool for sequence finishing.. Genome Res.

[38] Altschul SF, Madden TL, Schaffer AA, Zhang J, Zhang Z (1997). Gapped BLAST and PSI-BLAST: a new generation of protein database search programs.. Nucleic Acids Res.

[39] Carver TJ, Rutherford KM, Berriman M, Rajandream MA, Barrell BG (2005). ACT: the Artemis Comparison Tool.. Bioinformatics.

[40] Sullivan MJ, Petty NK, Beatson SA (2011). Easyfig: a genome comparison visualiser.. Bioinformatics.

[41] Alikhan NF, Petty NK, Ben Zakour NL, Beatson SA (2011). BLAST Ring Image Generator (BRIG): simple prokaryote genome comparisons.. BMC Genomics.

[42] Valle J, Mabbett AN, Ulett GC, Toledo-Arana A, Wecker K (2008). UpaG, a new member of the trimeric autotransporter family of adhesins in uropathogenic Escherichia coli.. J Bacteriol.

